# Bovine pericardial patch repair for abdominal aortic pseudoaneurysm to preserve lumbar arteries

**DOI:** 10.1093/icvts/ivac261

**Published:** 2022-10-31

**Authors:** Junya Nabeshima, Tomohiro Mizuno, Eiki Nagaoka

**Affiliations:** Department of Cardiovascular Surgery, Graduate School of Medical and Dental Sciences, Tokyo Medical and Dental University, Tokyo, Japan; Department of Cardiovascular Surgery, Graduate School of Medical and Dental Sciences, Tokyo Medical and Dental University, Tokyo, Japan; Department of Cardiovascular Surgery, Graduate School of Medical and Dental Sciences, Tokyo Medical and Dental University, Tokyo, Japan

**Keywords:** Aortic aneurysm, Bovine pericardium, Spinal cord injury

## Abstract

We describe a case of aortic repair using bovine pericardium for a pseudoaneurysm of a dissecting abdominal aorta. A 71-year-old man had undergone several aortic replacement surgeries for type B aortic dissection. He developed paraparesis after thoraco-abdominal aortic surgery and recovered. After 3 years, the scheduled computed tomography scan showed a pseudoaneurysm of the dissecting abdominal aorta. Because he was at high risk of spinal cord ischaemia, aortic repair using bovine pericardium was performed, and all lumbar arteries were preserved. During the 12-month follow-up, he was asymptomatic, and computed tomography scans showed no dilation of the aorta.

A 71-year-old man visited our outpatient clinic for a scheduled follow-up after multiple aortic surgeries. He had a history of Stanford type B aortic dissection 11 years before presentation and initially underwent descending aortic replacement 10 years ago. He had undergone ascending aortic replacement and thoraco-abdominal aortic replacement 3 years ago. Reconstruction of 3 pairs of intercostal arteries was attempted during the thoraco-abdominal replacement surgery; however, the dissecting aortic wall was too fragile to anastomose. The aortic patch of the intercostal arteries ruptured after anastomosis, and reconstruction was abandoned. He experienced paraparesis postoperatively but could walk unaided after months of rehabilitation.

Three years later, a scheduled computed tomography (CT) scan revealed a multilocular mass along the abdominal aorta (Fig. 1). Because the patient was allergic to iodine contrast agent, magnetic resonance imaging was performed revealing a pseudoaneurysm of the dissecting abdominal aorta. PET–CT showed no signs of infection.

**Figure 1:**
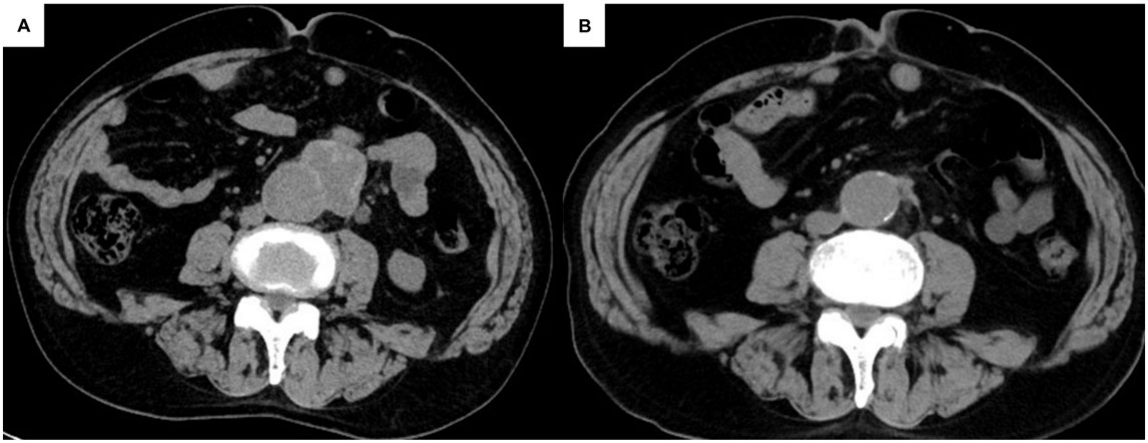
(**A**) Preoperative computed tomography image showing pseudoaneurysm of the abdominal aorta. (**B**) Postoperative computed tomography image showing repaired abdominal aorta without aneurysmal change.

We speculated that the patient was in an extremely high-risk situation for replacement surgery or endovascular stent graft insertion, which would sacrifice the lumbar arteries. To minimize the risk of paraplegia, it was important to preserve all lumbar arteries branching from the treated aortic range.

The abdominal aorta was exposed via midline laparotomy under general anaesthesia. A proximal clamp was placed on the thoraco-abdominal aortic graft, while distal clamps were placed on the bilateral common iliac arteries. The aneurysm was opened (Fig. 2). We detected 3 pairs of lumbar arteries: the left-side branches from the true lumen, the right branches from the false lumen (at the border between the true and the false lumen) and another detected in the right common iliac artery. The true lumen and a 1-cm wide part of the posterior adventitia were left for anastomosis, all lumber arteries were preserved, and all other intimal flaps and adventitia were resected. Almost two-thirds of the aortic wall was repaired with a bovine pericardial patch (model number 4700, Edwards Lifesciences LLC, Irvine, CA, USA). The pericardium was rectangular, and the width was adjusted for the aortic diameter. Another rectangular pericardium was sutured to the right common iliac artery, and the 2 pieces of pericardium were sutured. The length of the patch was 15 cm × 5 cm. After declamping, the pericardium was well inflated and the shape of the aorta was smooth and rounded (Fig. 2). Aortic clamp time was 74 min.

**Figure 2:**
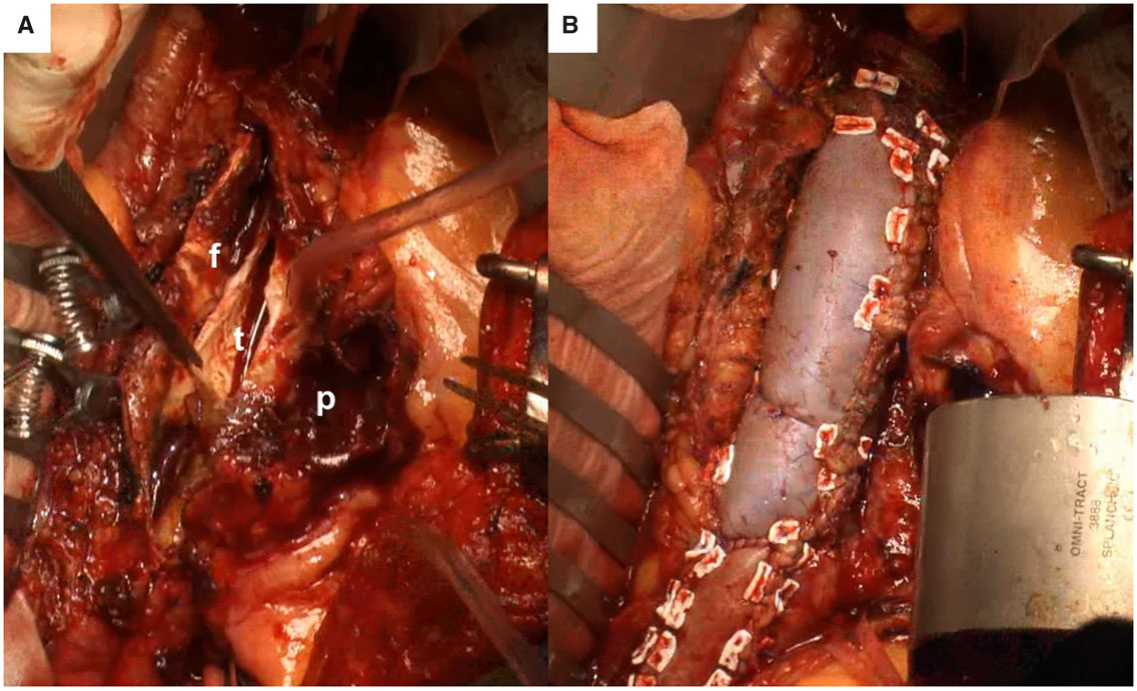
Intraoperative images. (**A**) Incised lumens before repair. (**B**) Repaired abdominal aorta using bovine pericardium. f: false lumen; p: pseudoaneurysm; t: true lumen.

Postoperative neurological disorders were not observed. The following day, slight paresis of the right lower extremity was detected that resolved shortly after physical treatment. Postoperative magnetic resonance imaging revealed that the abdominal aorta was well reconstructed without active problems. One year post-surgery, CT scans showed no dilation of the aorta, and the patient had no complaints regarding leg function.

## DISCUSSION

Abdominal aortic aneurysm is a risk factor for spinal cord injury in thoracic descending aortic operations [1]. In this case, we observed the opposite. The intercostal arteries had already been sacrificed, and the left subclavian artery and the remaining lumbar arteries were the only blood sources to the spinal cord. To avoid paraplegia, the lumbar arteries and other collateral circulation needed to be preserved.

Prosthetic grafts can be used for aortic replacement. However, reconstruction of each lumbar artery can be difficult due to stress on the anastomotic line of the dissected aortic wall. In this case, bovine pericardium was selected because its softness can help minimize the stress on the fragile wall of the dissected vessel. The key to this technique is to reproduce the shape of the aorta with little wrinkle. Therefore, we used separated 2 or more pieces of patch to fully conform to the vessel’s shape.

The use of the bovine pericardium for aortic repair has only been reported for the treatment of infectious diseases because of its anti-infective characteristics. The short-term outcomes are reported to be acceptable, but long-term outcomes are unclear [2, 3]. Careful follow-up is required to determine whether an aneurysmal change has occurred.

## CONCLUSION

We performed aortic repair using a bovine pericardium for the abdominal aorta to maintain lumbar arteries in a patient with a history of paraparesis after descending and thoraco-abdominal aortic replacement.

## Data Availability

The data underlying this article will be shared on reasonable request to the corresponding author. **Conflict of interest:** none declared.
